# Metronomic Chemotherapy for Metastatic Breast Cancer Treatment: Clinical and Preclinical Data between Lights and Shadows

**DOI:** 10.3390/jcm11164710

**Published:** 2022-08-12

**Authors:** Marina Elena Cazzaniga, Serena Capici, Nicoletta Cordani, Viola Cogliati, Francesca Fulvia Pepe, Francesca Riva, Maria Grazia Cerrito

**Affiliations:** 1 School of Medicine and Surgery, Milano-Bicocca University, 20900 Monza, Italy; 2 Phase 1 Research Centre, ASST Monza, 20900 Monza, Italy; 3 Oncology Unit, ASST Monza, 20900 Monza, Italy

**Keywords:** metronomic chemotherapy, breast cancer, safety

## Abstract

Metronomic chemotherapy (mCHT), defined as continuous administration of low-dose chemotherapeutic agents with no or short regular treatment-free intervals, was first introduced to the clinic in international guidelines in 2017, and, since then, has become one of the available strategies for the treatment of advanced breast cancer (ABC). Despite recent successes, many unsolved practical and theoretical issues remain to be addressed. The present review aims to identify the “lights and shadows” of mCHT in preclinical and clinical settings. In the preclinical setting, several findings indicate that one of the most noticeable effects of mCHT is on the tumor microenvironment, which, over the last twenty years, has been demonstrated to be pivotal in supporting tumor cell survival and proliferation. On the other hand, the direct effects on tumor cells have been less well-defined. In addition, critical items to be addressed are the lack of definition of an optimal biological dose (OBD), the method of administration of metronomic schedules, and the recognition and validation of predictive biomarkers. In the clinical context—where mCHT has mainly been used in a metastatic setting—low toxicity is the most well-recognised light of mCHT, whereas the type of study design, the absence of randomised trials and uncertainty in terms of doses and drugs remain among the shadows. In conclusion, growing evidence indicates that mCHT is a suitable treatment option for selected metastatic breast cancer (MBC) patients. Moreover, given its multimodal mechanisms of action, its addition to immunological and targeted therapies might represent a promising new approach to the treatment of MBC. More preclinical data are needed in this regard, which can only be obtained through support for translational research as the key link between basic science and patient care.

## 1. Introduction

Metronomic chemotherapy (mCHT) refers to the minimum biologically effective dose of a chemotherapeutic agent able to induce antitumor activity when given as a continuous dosing regimen with no prolonged drug-free breaks. Standard-of-care chemotherapy, instead, is based on administration of the maximum tolerated dose (MTD) of a drug(s) given for several cycles, with prolonged drug-free breaks between administrations. Since its inception, mCHT was perceived as a therapy to be used in very advanced or palliative settings, especially in those cancers, such as breast cancer, for which more complex and modern therapies are available and mistakenly considered more effective [1]. Despite the difficult circumstances in which mCHT took its first steps, it soon became evident that it had some peculiar properties, mainly related to its various mechanisms of action, and that these properties could be exploited to optimise the sequencing of treatment.

For clinical application, mCHT was first introduced in international guidelines in 2017. The International Consensus Guidelines for advanced breast cancer (ABC) stated that “*Metronomic ChT is a reasonable treatment option for patients not requiring rapid tumour response. The better studied regimen is CM (low-dose oral cyclophosphamide and methotrexate); other regimens are being evaluated (including capecitabine and vinorelbine). Randomised trials are needed to accurately compare metronomic ChT with standard dosing regimens*”. Clinical evidence gathered at that time, despite this being in early stages of development, supported a strong recommendation that the benefits outweighed the risks and harms even in the absence of randomised studies. A consensus on the published statement was reached by 88% of the panelists.

Since that point, mCHT became one of the strategies available for the treatment of ABC, even though the story of the treatment remained bedecked with lights and shadows. A significant concern is a lack of connection between laboratory data and the clinic: trial design has mainly been based on clinical outcomes without corresponding translational research aims or has been constrained by the very small number of patients enrolled. Improved linkage between preclinical and clinical research could reduce the gap between a correct diagnosis and the most favorable clinical outcome for the patient.

The purpose of the present review is to consider the lights and shadows of mCHT in preclinical and clinical settings, with the aim of highlighting both the positives and the missing aspects of this treatment approach.

With respect to preclinical research, we report data relating to the different mechanisms of action and the validation of some biomarkers, seeking to distinguish between established findings and work in progress, even though it is not always feasible to clearly demarcate these.

Regarding clinical research, among the lights, given the generally reported low incidence of severe side-effects related to mCHT, we decided to focus on these, together with quality-of-life (QoL) data, when available. Among the shadows, we reviewed study designs and end-points, and the presence of correlative biomarker studies, as these are areas requiring further exploration.

Considering the significant number of publications which relate to metronomic strategies in ABC patients, this review necessarily reports only key findings of research obtained in preclinical and clinical settings.

## 2. Materials and Methods

For the purposes of this review, we considered crucial studies with fully published results, which reported data concerning side-effects, QoL, symptom control or all of these. To better describe the toxicity of mCHT regimens, we included only studies that investigated pure metronomic regimens, in which drugs were administered at low dose, continuously, according to the definition of mCHT provided above [2]. We scanned databases using PubMed for keywords (e.g., *metronomic, breast cancer*), and, by using filters for the years *2010 to the present*), retrieved 247 results. We subsequently narrowed the field to clinical trials and randomized clinical trials, producing 64 results. The choice of selecting only papers published after 2010 was made due to the presence of different, and sometimes exhaustive, reviews published around that time, including one from our group. Amongst the clinical studies, we separately reported metronomic schedules with single or combination chemotherapy agents and metronomic schedules with chemotherapy agents combined with different targeted therapies, focusing on ABC. Our choice was informed by the lack of clinical trials in the early period, except for two which had enrolled a small number of patients.

For preclinical data, we conducted a literature search on PubMed and Web of Science using the terms “preclinical model of breast cancer and metronomic chemotherapy” and “mechanisms of action and biomarkers and metronomic chemotherapy” and selected the most relevant studies from 2000 to 2022. From the entire literature search, we retrieved 110 results, 67 of which were regarded as potentially relevant, 45 of which, in turn, were retained and fully reviewed.

## 3. Results

### 3.1. Preclinical Setting Lights

During the last twenty years, significant advances have been made in the understanding of the biology of cancer, including the interaction of tumour cells with their microenvironment. Several points of intervention for its treatment have emerged. For example, the importance and the mechanism of action of specific drugs have been highlighted, and it has also been understood that the way these molecules are administered—the dose and schedule—is very important [3], leading to the recognition of the importance of mCHT. Several mechanisms of action of mCHT have been identified, which include: (i) the prevention of tumour angiogenesis; (ii) direct effects on cancer cells; (iii) the induction of cellular senescence; and (iv) modulation of the immune system—with which the tumour cells interact directly—and of adjacent stromal cells. However, several challenges remain, particularly in terms of determining the mechanisms of action and identifying predictive biomarkers to identify those patients who will most benefit from mCHT.

We report what, in our opinion, can be considered “lights”—mechanisms and biomarkers that are well-documented for mCHT—and “shadows”, i.e., areas that require further research.

#### 3.1.1. Mechanisms of Action

Tumour-associated angiogenesis is defined as the sprouting of new micro-vessels from pre-existing ones; these new blood vessels can support tumour growth. However, neo-angiogenesis can also arise from cells recruited from the bone marrow, or that differentiate from cancer stem cells, in a process called “vascular mimicry” [4].

In recent years, research in this field has focused on understanding how different therapies act to prevent or block angiogenesis, mainly by inhibiting VEGF, which is the signal gradient towards which the growing vascular sprouts move. The anti-angiogenic effect of anticancer drugs at the MTD preferentially impacts proliferating endothelial cells (ECs), some of which participate in the germination of new tumour micro-vessels. Unfortunately, these effects are reversed during the two to three week pauses between subsequent treatments, as occurs with standard chemotherapy schedules. The anti-angiogenic stimulus of chemotherapy appeared to be improved when drugs were administered metronomically, in small doses, on a frequent schedule (daily, several times a week, or weekly), and continuously for prolonged periods, as shown in xenotransplant models, including for drug-resistant tumours [2]. Klement et al. established that orthotopic xenografts of human breast cancer responded significantly to continuous low-dose chemotherapy regimens when used in combination with a second anti-angiogenic drug, i.e., anti-VEGFR-2 antibodies, which led to inhibition of tumour angiogenesis and reduced tumour size [5].

To determine the sensitivity to angiogenic therapy of primary orthotopic breast cancer xenografts compared to distant metastases, Bridgeman and colleagues evaluated histological data, in which primary tumours exhibited intense angiogenesis while lung metastases did not. In the lungs, there was evidence of “vessel co-optation”, which is the capturing of existing vessels by tumour cells—particularly in a vascular-rich organ like the lung—migrating along the vessels of the host organ, thus supporting the metastatic process. These data confirmed the previous findings of Pezzella in 1996, who was the first to demonstrate the ability of cancer cells to enlist existing vessels to feed the growing tumour. Pezzella also suggested that the co-optation of the vessel constituted a mechanism of intrinsic resistance or a means of lowering the response to anti-angiogenic drugs.

Cancer cells, particularly those from highly metastatic tumours, are also capable of vasculogenic mimicry to escape anti-angiogenic therapy. Cancer cells can differentiate and enhance EC-like characteristics by expressing VE-cadherin and ephrin A2. This process is associated with increased tumour invasiveness and relapses [6].

Various preclinical studies have indicated that proliferating microvascular ECs represent the primary targets when tumours are treated with mCHT. Low-dose chemotherapy directly affects the tumour vessel through growth arrest and apoptosis of activated ECs. mCHT inhibits the expression of pro-angiogenic factors, such as VEGF, VEGFR2, bFGF, and SDF1, and induces the production of angiogenesis inhibitors, such as thrombospondin-1, in both stromal and cancer cells, platelet factor-4, and endostatin. Moreover, mCHT induces apoptosis in circulating ECs and inhibits endothelial progenitor cell (EPC) mobilisation [7,8]. In addition, combined treatment of anti-angiogenic and cytotoxic drugs synergistically hampered tumour progression and prolonged survival in tumour-bearing animal models [9,10]. An interesting study evaluated the molecular mechanisms of topotecan administered in mCHT mode, alone or in combination with pazopanib (an antiangiogenic tyrosine kinase inhibitor), in primary and metastatic orthotopic models of triple-negative breast cancer (TNBC); the impact of hypoxic conditions was also examined. The combination of metronomic topotecan and pazopanib significantly improved antitumor activity compared to monotherapy with both drugs, and prolonged survival, even in the context of advanced metastatic cancer, with important changes in tumor angiogenesis, tumor cell proliferation, apoptosis, HIF1α levels, and HIF-1 target gene expression [11].

Together, these data suggest that mCHT impacts on the altered blood flow of tumour vessels through their functional normalisation, rendering the delivery of anticancer drugs to the tumour more effective. Tumour vessel normalisation represents an emerging concept for mCHT-based tumour treatment.

The modulation of the immune system directly affects tumour cells and adjacent stromal cells [12] and the effect of chemotherapy on these components has been studied in different experimental settings. For example, the Kerbel group performed a preclinical study using an orthotopic model of syngeneic murine TNBC (EMT6/P) treated with the immune checkpoint inhibitor anti-CTLA4. The use of the monoclonal antibody partially inhibited tumour growth and this inhibitory effect was increased when the anti-CTLA4 antibody was combined with a low-dose cyclophosphamide (CTX) regimen, but not when the anti-CTLA4 antibody was combined with high-dose injection of CTX plus a low oral dose of CTX [13]. These results were further corroborated and expanded when the authors compared three different CTX protocols for anti-cancer efficacy in three murine breast cancer models. In an EMT6/P model, three different CTX regimens were studied: the MTD protocol, a low dose daily/continuous oral metronomic CTX, and medium dose intermittent CTX (CTX140 1q6d regimen) where the drug was injected in a medium dose, every six days. The latter protocol was more effective in inhibiting primary tumour growth than the MTD or continuous oral low daily dose CTX. In addition, CTX140 1q6d also produced anti-cancer results by stimulating the innate and adaptive immune systems. In fact, CTX140 1q6d upregulated PD-L1 expression on CD45+ and CD45− cells within the tumour microenvironment. Consistent with these preclinical data, a clinical study reported that therapy with an mCHT schedule in TNBC-patients expressing higher levels of PD-L1 in the tumour microenvironment resulted in better responses [14]. Orecchioni et al. investigated the effects of mCHT VNR, CTX and 5-FU, alone or in combination with checkpoint inhibitors, on the circulating immune cells of mice injected in the mammary fat pad with 4T1 TNBC cells. They found a synergistic effect in reducing circulating T, B, and NK cells when chemotherapy was given with anti-PD-L1. Notably, they observed that the reduction in the different immune cells triggered by mCHT in peripheral blood was not mirrored by a similar decrease in the intratumoral immune cell infiltrate [15].

**Figure 1:**
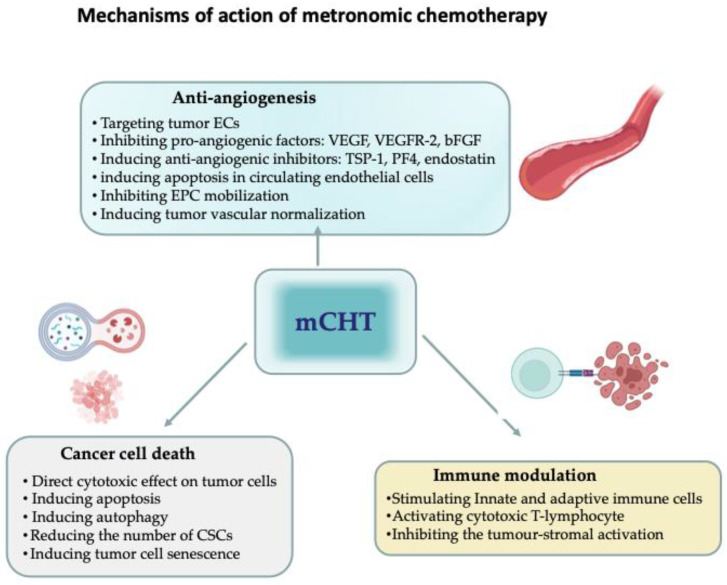
Mechanisms of action of metronomic chemotherapy (mCHT). The beneficial effects of mCHT are mediated by the inhibition of angiogenesis, the direct inhibition of tumour cell proliferation, and the stimulation of the immune system. Inhibition of angiogenesis plays a fundamental role in mCHT. The anti-angiogenic effects include direct inhibition of endothelial cells (ECs) proliferation via inhibition of pro-angiogenic factors, such as vascular-endothelial growth factor (VEGF), vascular endothelial growth factor receptor 2 (VEGF-R2), basic fibroblast growth factor (bFGF), and upregulation of endogenous angiogenic inhibitors, such as Thrombospondin 1 (TSP-1) endostatin, and platelet factor 4 (PF4), and inhibition of endogenous endothelial progenitor cells (EPCs) mobilization. Direct cytotoxic effects on tumor cells and decreased cancer stem cells (CSCs) population are also observed. mCHT also stimulates anticancer immunity by increasing cytotoxic activity of immune cell effectors and by inhibiting tumour-stromal activation. Figure created with BioRender.com.

Figure 1 describes the main mechanisms of action of mCHT.

#### 3.1.2. Biomarkers

In the last decade, considerable efforts have been made in the preclinical setting to identify valuable biomarkers that could be used to stratify breast cancer patients and monitor the effectiveness of the mCHT regimen [16,17]. Potential biomarkers have been studied to demonstrate, for example, its anti-angiogenic action. They include circulating blood biomarkers, such as VEGF, angiopoietin, thrombospondin ½(TSP-1/2) and circulating ECs [7,18], polymorphisms of a single nucleotide (e.g., VEGF, IL6, IL8) [19], and immunohistochemistry (e.g., VEGF and TSP-2). Techniques applied have also included functional imaging (e.g., dynamic contrast-enhanced magnetic resonance imaging or dynamic contrast-enhanced computed tomography) [20] and laser speckle flowmetry to evaluate the efficacy of mCHT CTX treatment in tumor shrinkage and tumor vasculature response [21]. More recently, it has been shown, in a preclinical study of patient-derived xenografts (PDXs) from CDK12HIGH and CDK12LOW human breast cancers, that CDK12 overexpression can predict response to metronomic methotrexate-based therapy. In addition, a retrospective analysis of lymph-node-positive breast cancer patients, randomized to receive metronomic cyclophosphamide plus methotrexate (CM) chemotherapy or no chemotherapy after completion of standard adjuvant treatments, confirmed CDK12 expression as a valuable biomarker in breast cancer patients [22].

### 3.2. Preclinical Setting—Shadows

#### 3.2.1. Mechanisms of Action

Preclinical and clinical evidence suggests a direct effect of mCHT on cancer cells [1]. For instance, it has been shown that protracted exposure to paclitaxel (PTX)—a taxane usually administered to treat breast cancer—induced a stronger cytotoxic effect than a short exposure, indicating that dose and duration are essential factors in the anticancer activity of PTX in human cancers, and prolonged exposure might increase this effect [12]. Recently, Roy et al. documented the efficacy of methylglyoxal, a highly reactive glycolytic metabolite, on breast cancer stem cells, and that its combination in an mCHT schedule with doxorubicin or cisplatin enhanced cytotoxicity towards MCF-7 and MDA-MB-231 cell lines [23].

Salem et al., demonstrated that low doses of the muscarinic agonist carbachol, combined with PTX, reduced MCF-7 cell growth in vitro, likely via down-regulation of the cancer stem cell population [24]. Recently, we confirmed that the mechanism of action of anticancer agents, such as 5-fluorouracil (5-FU) and vinorelbine (VNR), can be significantly different when given metronomically compared to administration following an MTD schedule [25]. We showed that, in TNBC cells, metronomic combinations of VNR and 5-FU could inhibit cell growth by inducing apoptosis and autophagy, and by significantly increasing cellular senescence [25]. Many studies have demonstrated effects of senescence, which may be involved in cancer prevention but also in its aggressiveness. There is both light and shadow with respect to the role of senescence in cancer. Additional studies are necessary to better understand the role of senescence in cancer and assess whether it is beneficial or detrimental for patients.

Considering the different effects of the combinatorial regimens of mCHT, it is reasonable to assume that further mechanisms remain to be discovered.

#### 3.2.2. Biomarkers

Several biomarkers have been studied in TNBC to identify new actionable targets [26]. Among them FGFR1 amplification (the most frequent aberrancy implicated in tumorigenesis) was found in 18–33% of samples. Notably, antibodies against the different FGFR isoforms are already being tested in different preclinical settings with good efficacy. Therefore, the combination of anti-FGFR antibodies with mCHT might also be envisioned for the future. Remarkably, it has already been shown in preclinical studies on TNBC cells that when PD-1 mAbs are combined with metronomic-PTX treatment, the efficacy of anti-PD-1 is improved. These studies attempted to respond to a clinical need, i.e., the unsatisfactory effect of PD-1/PD-L1 monoclonal antibodies used alone [27]. In another study, the authors analyzed the immunomodulatory effects of EphA2-ILs-DTXp, a targeted nanoliposomal taxane, in combination with checkpoint inhibitors. They found, in an EMT-6 breast cancer model, that metronomic dosing of docetaxel improved tumour growth suppression by increasing the activity of CD8+ T cells [28]. Earlier, Francia et al. showed, in Her-2-positive human metastatic breast cancer xenografts, that the efficacy of trastuzumab was enhanced when combined with metronomic low-dose CTX [29]. Recent preclinical studies have used CTX combined with other drugs to treat breast cancer with varying success [30].

Significant attention has also been paid to the identification of non-invasive imaging markers of response to chemotherapy treatment [31]; for example, therapy-induced responses, including apoptosis and proliferation, have been traced in preclinical cancer models using label-free optical imaging techniques, such as spatial frequency domain imaging [32].

Another important aspect that deserves attention concerns the optimal biological dose (OBD) of drugs, defined as the smallest effective dose that causes the highest tumour volume shrinkage with no or minimal toxicity. Several preclinical models have been used to evaluate the toxicological and biological effects of treatments, although none is considered perfect for determining drug OBD. For this reason, to obtain accurate results, it is important to select the most suitable preclinical model to study. In a pioneering paper, Shaked and colleagues [33] identified the OBD of various mCHT regimens in four different preclinical cancer models, including breast cancer. Each OBD obtained by the authors was associated with the highest reduction in circulating VEGFR2-positives

Critical issues remain to be addressed regarding the administration of metronomic schedules and the recognition and validation of predictive biomarkers. These aspects were underlined in a recent study [34] in which the authors highlighted the importance of the pharmacokinetic effects of mCHT, which are often overlooked, even though they are of fundamental significance, considering the implications of this understanding for both preclinical research and the design of clinical trials.

Overall, solid and reliable biomarkers (i.e., diagnostic, predictive) are still needed to predict which patients are more likely to benefit from mCHT.

**Figure 2:**
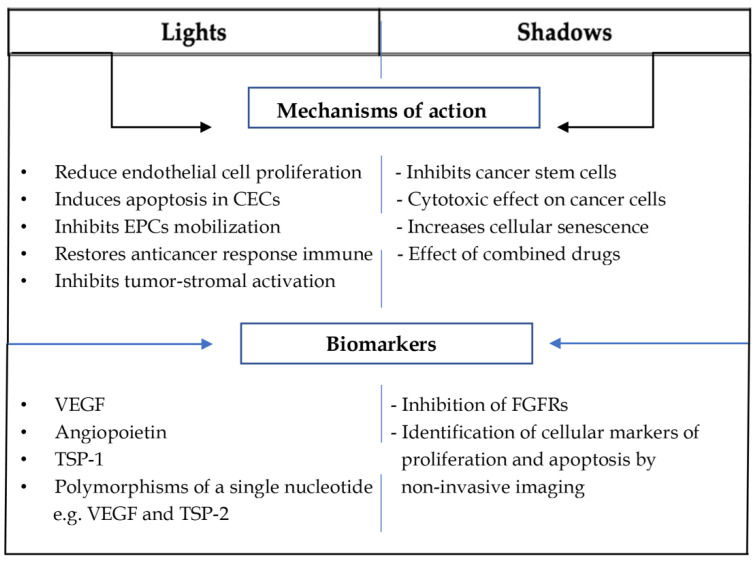
**The scheme summarises the mechanisms of action and biomarkers that have been determined (highlights) and those that need further investigation (shadows) related to mCHT**. Circulating endothelial cells (CECs); endothelial progenitor cells (EPCs); vascular endothelial growth factor (VEGF); thrombospondin-1 and -2 (TSP-1, and TSP-2); fibroblast growth factor receptors (FGFRs).

A summary of the mechanisms of action and biomarkers that have been established (lights) and those that need further investigation (shadows) related to mCHT are outlined in Figure 2.

### 3.3. Clinical Setting—Lights

Breast cancer was the first type of tumour investigated for application of mCHT. In patients with ABC, the main goals remain QoL improvement and disease symptom reduction, rather than tumour response. In this context, mCHT provides an excellent alternative to conventional chemotherapy, especially considering the low incidence of side-effects. The most studied drugs are CTX, methotrexate (MTX), VNR and capecitabine (CAPE), alone, or combined with other chemotherapy agents or targeted therapies.

#### 3.3.1. mCHT Alone

As highlighted in different reviews, CTX was the first and is the most studied drug, usually administered at a continuous dose of 50 mg/day. The metronomic dose used in several clinical trials was set, on an empirical basis, to be 50 mg p.o. daily [35]; subsequent steps included, first, the addition of MTX [24] and, subsequently, of biological agents.

Different trials [36,37,38] have studied metronomic CTX in ABC patients, mostly in those heavily pre-treated using several lines of treatment. Overall, Grade 3 leukopenia and neutropenia, ranged between 3–10% of patients, whereas Grade 4 was reported in approximately 4% of patients.

A retrospective analysis [36] of 61 patients with endocrine-resistant ABC who already received two lines of chemotherapy treated with oral CTX at the dose of 50 mg/day (Cohort 1, N = 22) or CTX at the same dose, together with MTX 2.5 mg orally twice a week (Cohort 2, N = 39), reported Grade 3 leukopenia in 5% of the cases treated with CTX alone and 3% of cases in those treated with CTX + MTX. All other toxicities, such as nausea/vomiting, mucositis and diarrhoea were Grade 1 or Grade 2. Symptom control was achieved in 54% of the cases.

Similar results were described by Lu et al. [39], who retrospectively reviewed data regarding the efficacy and safety of CTX+MTX in 186 ABC patients. The authors reported a very good safety profile for this combination; the incidence of any grade of leukopenia and neutropenia was 0.6%. Other toxicities, specifically, nausea and AST/ALT elevation, which were mostly related to MTX, were below 10% (7.1% and 3%, respectively).

The combination of CTX with etoposide was explored by Mutlu et al. [38] in a retrospective analysis of 77 heavily pre-treated ABC patients. The patients received continuous oral CTX at a dose of 50 mg/day and oral etoposide given as 50 mg twice a day for two days per week. The toxicities related to mCHT were low and mainly haematologic: G3 and G4 leukopenia were reported in 10.4% and 3.9% of the cases, respectively, and G3 and G4 thrombocytopenia in 2.6 and 0% of cases, respectively. No Grade 4 emesis was observed. In this study, the addition of etoposide, a drug with well-known haematologic toxicity, slightly increased the incidence of haematologic events, which was usually very low, as described above.

Another much-studied drug is VNR, alone, or, most commonly, in combination with CAPE, CTX, or both. Despite the availability of Phase 1 trial data [40], which, for VNR, suggested a recommended dose of 50 mg thrice a week when used as a single agent, some trials tested alternative schedules, such as 30 mg/day, without interruptions. This schedule was studied in a multicentre, open-label, single-arm study which enrolled nine patients and was closed early due to one Grade 5 toxicity (febrile neutropenia) [41].

Many more studies have explored the combination of VNR with CAPE, CTX, or both [42]. One of the most extensive studies conducted considering combinations of different metronomic drugs was that undertaken by Montagna et al. [42]; a total of 43 patients in a naïve group and 65 in a pre-treated group received 40 mg VNR thrice a week plus 500 mg CAPE thrice a day, together with CTX at a dose of 50 mg/day (VEX), administered continuously. Among the considerable strengths of this study was the prospective design, enabling corroboration of evidence that the incidence of side-effects with mCHT is very low; the most frequent G3 treatment-related adverse events were neutropenia (5%), increase in transaminases (5%) and hand and foot syndrome (7%).

Similarly, the XeNa trial [43] reported a lower incidence of some severe adverse events in the metronomic group compared with the standard regimen (fatigue: 9.7% vs. 17.2%). This was a large, randomised Phase 2 study that enrolled 120 Her2- ABC patients; the patients were randomised to receive either the standard schedule of VNR (60 mg/m^2^ day 1 + day 8 in the first cycle, followed by 80 mg/m^2^ day 1 + day 8 in the following cycles) or metronomic VNR 50 mg three times a week. Capecitabine 1000 mg/m^2^ twice a day for days 1–14 was administered in both groups.

Finally, Krajnak et al. [44] retrospectively analysed 35 MBC patients treated with a combination of CTX 50 mg daily and MTX 2.5 mg every second day; discontinuation due to adverse events occurred in 9% of the patients, whereas only 11% of the patients stopped MTX, mainly due to gastro-intestinal toxicity.

Our own research on mCHT has comprised different studies over the years, starting with the VICTOR-1 study [45], which established the maximum tolerated dose (MTD) of VNR as 40 mg thrice a week in combination with fixed doses of CAPE (500 mg thrice a day); these results were subsequently confirmed in the multicentre VICTOR-2 study [46], and reinforced in the VICTOR-6 real-world study [47]. In all these studies, we only observed mild (Grade 1–2) toxicities (Grade 1–2: nausea/vomiting 15.4%; hematologic effects 14%, diarrhoea 12%), even in the large population analysed in the VICTOR-6 trial (600 patients), or subgroups of patients, such as those aged over 75 years.

Keeping in mind that the main aim in the treatment of ABC patients is the improvement of OS, the duration of the clinical benefit produced by a defined regimen is a crucial element: Montagna et al. [48] recently analysed data for their cohort of ABC patients enrolled in a clinical trial of mCHT (VNR + CTX + CAPE), reporting a PFS rate at three years of 25.4%. The main Grade 3–4 adverse event observed was hand-foot syndrome (7%), with no evidence of specific or more important, cumulative or delayed toxicities with mCHT, which was confirmed as a very well-tolerated strategy.

#### 3.3.2. mCHT in Combination with Targeted Agents

Many other authors have investigated the combination of CTX + MTX with other anti-cancer agents, such as the VEGFR inhibitor vandetanib (VAN) [49], and non-anticancer drugs, such as thalidomide [50], dalteparin and prednisone or idiotype vaccine; however, outcomes showed non-significant therapeutic improvements. Due to its safety profile and the ease of administration, mCHT has been studied in combination with different targeted agents, mainly anti-Her2, anti-VEGF or tyrosine-kinase inhibitors (TKIs). Although the combination with targeted agents has led to a renewed interest in mCHT, the side-effects related to biological drugs partially cancel the significant advantage in terms of toxicity provided by this modality of administration. Nevertheless, these combinations represent an alternative to CHT standard-dose regimens and are of especial value for frail or elderly patients.

*mCHT in Combination with Anti-HER2 Agents*

The largest and most well-conducted study regarding the combination of mCHT with anti-HER2 agents was carried out by Wildiers et al. [51] in frail ABC patients, defined by age (70 years or older and 60 years or older) but without the presence of comorbidities. Patients were randomized to receive metronomic oral CTX 50 mg per day plus trastuzumab and pertuzumab, or trastuzumab and pertuzumab alone at standard doses. The most frequent grade 3–4 adverse events in the mCHT arm were hypertension (12%), diarrhoea (12%), dyspnoea (10%), fatigue (5%), pain (5%), and a thromboembolic event occurred in 10% of the patients. CTX discontinuation was necessary for 22 patients (54%). Grade 3 adverse events were heart failure (5%), diarrhoea, fatigue, pain and anorexia (5% for each of the four adverse events). The incidence of these events was very similar to that reported in the control arm and were more likely to have been related to the anti-HER2 agents rather than mCHT. This study was one of the few that analysed patients’ quality of life [52]. The authors assessed HRQoL using the EORTC QLQ-C30 and the EORTC Elderly specific module QLQ-ELD14 at baseline, and weeks 9, 27, and 52. The primary HRQoL domains were global health status/QoL scale (GHQs), fatigue and pain. No statistically significant differences in terms of HRQoL domain changes were detected over time between the two treatments.

Other authors [53] investigated the combination of mCHT (VNR 40 mg thrice a week), in combination with trastuzumab, reporting very few adverse events, the most important being neutropenia, which was observed in 10% of the patients.

*mCHT in Combination with Anti-Angiogenic Drugs*

The combination of mCHT with anti-angiogenic agents is probably one of the most promising associations, particularly when CTX is part of the regimens, given that its peculiar anti-angiogenic properties have been demonstrated in preclinical [13] and clinical settings [54,55,56,57]. As already illustrated above, mCHT inhibits circulating ECs and EPCs, and modulates pro- and anti-angiogenic molecules, such as VEGF, thrombospondin-1 and VEGFR2, amongst others [58].

Different drugs have been tested in combination with bevacizumab (the monoclonal antibody against VEGF-A), with CTX being the most used at a dose of 50 mg/day, in combination with either CAPE [54,56] or MTX [55]. Grade 3 or 4 adverse effects that occurred in the trials were hypertension, leukopenia, neutropenia and transaminitis. One study [54] explored, together with efficacy, the relationship among circulating ECs and circulating EPCs and the response and outcomes of the patients.

Similar toxicities were observed in a Phase III study which randomized ABC patients to receive bevacizumab with either PTX (arm A) or daily oral CAPE-CTX (arm B, CAPE 500 mg x 3/daily + CTX 50 mg/day) as a first-line treatment [56]. A major strength of this study was the direct comparison between mCHT and standard chemotherapy in terms of QoL evaluation, which considered physical well-being, measured by self-assessment questionnaire. The questionnaire included indicators of physical well-being, mood, coping effort, overall treatment burden, health perception, appetite, tiredness, hair loss, nausea/vomiting, and numbness/tingling in the hands/feet. The incidence of primary endpoint-defining adverse events was similar between the two arms (25% vs. 24%; *p* = 0.96); 17 patients stopped treatment because of unacceptable toxicities, 12 (17%) in the standard CHT arm and 5 (7%) in the mCHT arm. Although the study failed to meet its primary endpoint, it provided important information regarding the QoL of ABC patients treated with mCHT; these reported substantially less hair loss (*p* < 0.0001) and less numbness with increasing time (*p* < 0.01) than those treated with standard CHT, and a tendency toward a lower overall treatment burden (*p* = 0.11).

Considering that different mechanisms account for the anti-angiogenic effect of mCHT, such as the selective inhibition of proliferation and/or induction of apoptosis of activated endothelial cells, the selective inhibition of endothelial cell migration, increase in the expression level of TSP-1 and sustained decrease in the levels and viability of bone marrow-derived EPCs, other authors have tested mCHT in combination with celecoxib, sorafenib, and vandetanib.

Together these studies indicate that the combination of mCHT and anti-angiogenic agents is feasible, with a slight increase in toxicities, mainly related to the anti-angiogenic drug; in our opinion, what is missing in this area is a systematic, prospective collection and analysis of biomarkers, which could definitively validate the principal mechanisms of action of mCHT.

*mCHT in Combination with other Targeted Agents*

Different combinations of mCHT have been explored to date including with celecoxib [59,60]), sorafenib [61], veliparib [62,63] and some others.

Perroud et al. postulated that the high expression in BC of the prostaglandin synthase enzyme cyclooxygenase-2 (COX2) could be a reasonable basis to combine metronomic CTX with the anti-COX2 agent celecoxib. These authors also evaluated many different biomarkers of angiogenesis, such as VEGF, TSP-1 and others, as well as QoL. Together with an excellent safety profile, specifically, the absence of G3 adverse events, the authors, when investigating biomarkers of angiogenesis, observed a decrease in VEGF concentration, whereas TSP-1 did not change significantly over the period, nor did the percentage of circulating EPCs and ECs. The authors also evaluated quality of life using the FACT-B questionnaire, reporting a marginally significant increase in functional well-being and a significant increase in additional concerns.

The same authors [60] investigated the combination above in a larger set of ABC patients, confirming the toxicity profile.

Another interesting combination of letrozole 2.5 mg/day plus CTX 50 mg/day and sorafenib every fifth day at a dose of 400 mg bid was explored as neoadjuvant treatment in a cohort of 13 early BC patients [61]. These authors also evaluated the expression of some biomarkers, such as VEGF-A and CD31, reporting a significant reduction at Day 14 and at definitive surgery in comparison to baseline. In contrast, a reduction in VEGF-A expression was detected only when comparing levels at the time of surgery with baseline. The authors also demonstrated that the concentration of sorafenib was not affected by dosing in combination with CTX, whereas mean plasma concentrations of CTX were significantly lower following concomitant administration of sorafenib and letrozole compared with concomitant administration of letrozole alone. The toxicity of this combination was notable and would be incompatible with clinical practice. The advantages of this study included the comprehensive evaluation of different parameters, such as ^18^FDG-PET changes over the time, which showed a significant reduction in SUV uptake at the surgery in comparison to baseline in all patients and for all biomarkers (i.e., CD31 significantly suppressed; VEGF-A expression significantly suppressed in response to treatment).

Finally, one of the most intriguing combinations tested is that of mCHT with poly [ADP-ribose] polymerase (PARP) inhibitors (PARPi). The rationale for this combination relies on the activity of PARPi in mutated BRCA patients, which can increase the DNA damage caused by chemotherapy; moreover, considering that these agents are administered orally, they could be paired with mCHT in an all-oral regimen.

Veliparib was investigated in combination with CTX in two different studies, one of which was a Phase 1 trial, [62,63], in different cohorts of heavily pre-treated TNBC patients. Overall, the combination did not improve the activity of CTX as a single-agent in the comparison study, though toxicity was acceptable and Grade 3–4 event percentages were below 10%.

Considering the high potential of combining low-dose, oral therapies with subcutaneous formulations of some anti-HER2 agents (e.g., trastuzumab, pertuzumab/trastuzumab), with novel and more potent oral TKIs, such as neratinib or tucatinib, or with novel therapies, further studies should be undertaken which aim to clarify: (1) the best mCHT backbone for targeted therapy; (2) the most appropriate drugs to enhance the biological activity of chosen interventions; and (3) which biomarkers best reflect the biological effects of drugs used in metronomic regimens. It would be beneficial for these future studies to include QoL evaluation, patient adherence, and biomarker evaluation. In our opinion, it is scientifically meaningless to continue to see published small, mostly retrospective, collections of cases, instead of joining forces to design a prospective, academic study with well-defined aims and end-points (including head-to-head comparisons, prospective QoL evaluation, biomarker assessment, etc).

Table 1 summarises the toxicities observed in the main studies.

As described in a recent review published by our group [1], this excellent toxicity profile is associated with well-documented clinical activity in various types of cancer and in ABC patients.

In a recently published systematic literature analysis [65], Lien et al. reviewed the status of knowledge regarding mCHT across different types of cancer. They reported that the main cancer types included in mCHT trials were breast (26.25%) and prostate tumours (11.25%) and the main agent was CTX (43%). Differences in terms of adverse event reporting were noted. Most of the studies used the NCI-CTCAE (any version) criteria, while seven used the WHO criteria. The authors emphasized that, despite the differences in reporting, mCHT was found to lead to low toxicity rates; in particular, no toxicity affected more than 6% of all pooled patients. The availability of such a systematic literature review compensates, to some extent, for the high heterogeneity observed in mCHT trials, at least with respect to toxicity.

Finally, it is our opinion that one of the most important lights of mCHT was its role during the COVID-19 pandemic. Even if the pandemic no longer represents an urgent emergency for public health services, we believe that it has provided an important lesson for the entire healthcare system, which should probably be reorganised in some areas to address future emergencies. In this context, the low toxicity profile of mCHT, together with its documented efficacy in some cancer types, above all ABC, should enable physicians to continue to treat patients in settings other than hospitals, such as the home, reducing admissions to hospital as much as possible.

### 3.4. Clinical Setting—Shadows

The prominent shadows regarding the use of mCHT in ABC patients include the different regimens and schedules applied, even when the same drugs are used, the small number of patients enrolled in the different trials, the retrospective design of most studies, and the almost complete absence of prospective randomized trials. The only randomized trial available to date has been the XeNa trial, which has some limitations, above all, the study design, which was not set up to provide a direct comparison between the two arms, and the use of a non-metronomic schedule for CAPE, which affected the toxicity results obtained. These are only some of the issues that require to be addressed to ensure that mCHT can be applied as a therapeutic strategy.

The need for an empirical basis for determining the optimal OBD and in monitoring the therapeutic activity of the drugs used in a metronomic schedule is a crucial point; for those drugs, such as CTX which is associated with a precise and known effect (angiogenesis), the OBD can be easily determined by assessing the maximum reduction in viable peripheral blood circulating VEGFR-2 or EPCs [66]. Monitoring of VEGFR-2 blood levels during treatment can also be easily achieved, as a useful dynamic biomarker to evaluate the response. Unfortunately, not all the drugs used in the different metronomic schedules are clearly associated with a precise mechanism of action, and not all the mechanisms are evaluable by a specific biomarker. For example, the well-known effect on T-reg down regulation observed with some metronomic schedules is only a partial and incomplete expression of the immune system stimulation.

In our opinion, the main limitation remains the absence of randomised studies for the vast majority of combinations and settings: fortunately, some trials [60] have filled this gap and some others are due, such as TEMPO BREAST (EudraCT number 2014-003860-19), which compared iv vs. a metronomic oral formulation of VNR as the first-line of treatment in 164 HR + /HER2-ABC patients randomly assigned to one of two treatment arms, and METEORA (NCT02954055), a multi-center, randomised phase II trial that randomised women with ER-positive, HER2-negative (human epidermal growth factor receptor 2-negative) metastatic or locally relapsed breast cancer in a ratio of 1:1, to receive a metronomic regimen of VNR plus CTX and CAPE, or the conventional paclitaxel monotherapy, the results for which are awaited soon.

Another important shadow is the almost total absence of QoL evaluation, even in trials that enrolled large populations of ABC patients; this has been performed sporadically but, unfortunately, was not planned for in most of the prospective studies.

## 4. Discussion

mCHT for the treatment of breast cancer treatment started slowly at the beginning of the 2000s; at that time, most physicians believed that it was nothing more than a palliative therapy, confining its use to very late stages in care.

With increasing evidence coming from scientists all around the world who tested mCHT in single arm, proof-of-concept studies in cancers different from breast (e.g., lung, prostate, paediatric, head and neck and ovarian cancers, glioblastoma), it became evident that mCHT could play a different role.

Even after many years of clinical use, especially as part of treatment of ABCs, the use of mCHT remains a matter of debate in the scientific community. Physicians are divided between those who have adopted it in their clinical practice, based mainly on its safety profile, and those who remain fierce opponents, citing many different reasons. Research is required to look deeper into developing strategies to improve the efficacy of drugs and significantly reduce toxicity.

In this paper, we have reviewed the available data regarding mCHT in ABC patients, seeking to highlight the lights and shadows of this strategy. Among the “lights” of mCHT, we considered the safety profile of the different drugs as options for treatment of ABC patients, alone or in combination with targeted agents, and the data regarding QoL. As well covered in the literature analysis by Lien et al. [65], despite differences in reporting, mCHT has been found to lead to low toxicity rates, in particular no toxicity affected more than 6% of all pooled patients. This represents, in our opinion, the brightest light of mCHT.

In the preclinical setting, we evaluated as “lights,” the well-described mechanisms that prevent or block angiogenesis, mainly by inhibiting VEGF and, to some extent, the effect of mCHT immunomodulation.

Among the “shadows”, we discussed the absence of prospective randomised trials, the design and the end-points adopted in some studies, and the heterogeneity of existing schedules, even for the same drug. Recently, Mishra-Kalyani et al. considered the role of control arms in oncology [67]; they suggest that, even though randomised control trials allow for a comparison of treatment arms with minimal concern for confounding by known and unknown factors, in some situations, and for some strategies, a randomised study is not feasible. These authors suggest that, when such designs are not possible, the incorporation of external control data into the study design could get around the obstacle. In the absence of the possibility of conducting prospective randomised phase 3 studies in different settings of BC and for different populations (TNBC, Luminal, HER2 + ve), we hope that this suggested shortcut will allow a definitive comparison between standard and metronomic strategies.

From the preclinical point of view, the “shadows” are represented by all the aspects still to be clarified, including the different factors that might facilitate the use of combinatorial regimens of mCHT. These include understanding of the mechanisms of action of metronomic schedules and the recognition and validation of predictive biomarkers, which, if identified, would provide valuable support for clinical application.

Our aim is to encourage readers to extend the amount of available data collected on mCHT for the treatment of ABC patients and, perhaps, to push them to consider this strategy in their clinical practice for some patients, in some emergency situations, or in low- or middle-income countries, where a low-cost strategy, such as mCHT, might partially rebalance disparities.

## 5. Conclusions

The efficacy of other agents in mCHT treatment combined with antiangiogenic treatment should be investigated. An example of a promising approach is the use of Bruton’s tyrosine kinase (BTK) inhibitors, such as ibrutinib, which was developed to treat several blood cancers, but has recently been shown to act effectively in solid tumours [68,69]. These drugs hit multiple targets and are associated with numerous expected outcomes. It has already been shown that ibrutinib has an antimetastatic effect on MCF-7 cells by activating the MAPK/NF-kB/AP-1 pathway and inhibiting MMP-9 expression [70]; reducing the viability of Erb2-positive (Erb2+) breast cancer cell lines by inhibiting the phosphorylation of receptor tyrosine kinases ErbB1, ErbB2, ErbB3 [71]; inhibits xenograft tumour growth by decreasing HER2, BTK, Akt, Erk, and increasing cleaved caspase-3 [72]; and affects anti-tumour immunity by reprogramming myeloid-derived suppressor cells (MDSCs) to mature DCs thus preventing tumour growth and metastasis, as demonstrated in a murine model of breast cancer [73].

Preclinical models of breast cancer could be developed to gain a more in-depth understanding of the basis of resistance, relapse, or progression of mCHT therapy, as well as to perform precise therapeutic tests of mCHT treatment by using mice with metastatic disease or mice carrying patient-derived breast cancer tissue, to characterize and improve the efficacy of antitumor combination therapies in vivo, as suggested by Kerbel [74].

From this extensive analysis focused on lights and shadows, we can conclude that mCHT *per se* is not associated with severe toxicities, especially haematological or gastro-intestinal effects, as detailed in the studies reported in this review, and as described in other similar papers [75,76]. However, mCHT loses this advantage, which is peculiar to this method, when different agents, with their related toxicities, or other chemotherapy drugs administered at standard doses, are co-administered [43,59].

Considering that improvement in the QoL of ABC patients, by reducing treatment toxicity, is one of the main goals of the Global Alliance Against Cancer, we strongly believe that current strategies, such as immunotherapy, should be studied in association with mCHT.

Global efforts should be combined so as not to throw away such a precious amount of data. More modern study designs should be used to clearly demonstrate if mCHT should be incorporated into our current strategies for treatment.

## Figures and Tables

**Table 1:** Summary of toxicities observed in the main studies.

Author (Year)	Regimen	Toxicity Grade 1–2 ^1^	Toxicity Grade 3–4 ^1^
Krajnak (2021) [41]	VNR 30 mg/day, continuously	Increased AST/ALT 22%	Febrile neutropenia (Grade 5)
Wang (2021) [53]	VNR 40 mg 3/week + trastuzumab 6 mg/kg (loading dose)	Nausea 15% Leukopenia 15% Increased ALT/AST 15% Diarrhoea 10% Peripheral neuropathy 10%	Neutropenia 10%
Brems-Eskildsen (2020) [43]	Arm A: VNR 60 mg/m^2^ day 1 + day 8 in the first cycle followed by 80 mg/m^2^ day 1 + day 8 + CAPE 1000 mg bid × 14 days, Q21 Arm B: VNR 50 mg 3/week + CAPE 1000 mg bid × 14 days, Q21	Arm A vs. Arm B Diarrhoea 53.2% vs. 46.5% Nausea 43.5% vs. 32.7% Mucositis 40.3% vs. 41.4% Fatigue 32.2% vs. 29.3% Hand-foot 48.4% vs. 44.8% Constipation 29% vs. 29.3% Neuropathy 29% vs. 25.9% Neutropenia 25.8% vs. 24.1% Dyspnea 24.2% vs. 18.9% Increased ALT/AST 20.9% vs. 17.2% Joint affection 19.4% vs. 17.2% Pain 19.3% Leucopenia 16.1% vs. 13.7% Fever 14.5% vs. 18.9% Abdominal pain 14.5% vs. 17.2% Back pain 11.3%	Fatigue 9.7% vs. 17.2%
Cazzaniga (2019) [64]	Different schedules	Nausea/vomiting 15.4% Hematologic 14.0% Diarrhoea 12% Fatigue 10.3%	<10%
Montagna (2022) [48]	VNR 40 mg 3/week + CTX 50 mg/day + CAPE 500 mg 3/day	Not reported	Hand-foot syndrome 7%
Perroud HA (2013) [59]	CTX 50 mg/daily + celecoxib 200 mg bid	Leucopenia G1 13.3% Neutropenia G1 6.7% Thrombocytopenia G1 6.7% Neutropenia G2 13.3% Anaemia G2 26.7%	None
Bazzola (2015) [61]	Letrozole 2.5 mg/day + CTX 50 mg + sorafenib 400 mg bid every 5th day	Alopecia 76.9% Neutropenia 38.5% Sensory neuropathy 38.5% Weight loss 38.5% Hand-foot syndrome 30.7% Fatigue 30.7% Rash 30.7% Dehydration 30.7% Anorexia 30.7% Arthralgias 23% Joint function 23% Hypertension 15.4% Mucositis 15.4% Acne 23%	Hand-foot syndrome 69.3% Rash 69.3% Diarrhoea 46.1% Dehydration 23%

^1^ Reported if incidence > 10%.

## References

[1] Cazzaniga M.E., Cordani N., Capici S., Cogliati V., Riva F., Cerrito M.G. (2021). Metronomic Chemotherapy. Cancers.

[2] Hanahan D., Bergers G., Bergsland E. (2000). Less is more, regularly: Metronomic dosing of cytotoxic drugs can target tumor angiogenesis in mice. J. Clin. Investig..

[3] Browder T., Butterfield C.E., Kraling B.M., Shi B., Marshall B., O’Reilly M.S., Folkman J. (2000). Antiangiogenic scheduling of chemotherapy improves efficacy against experimental drug-resistant cancer. Cancer Res..

[4] Wechman S.L., Emdad L., Sarkar D., Das S.K., Fisher P.B. (2020). Vascular mimicry: Triggers, molecular interactions and in vivo models. Adv. Cancer Res..

[5] Klement G., Huang P., Mayer B., Green S.K., Man S., Bohlen P., Hicklin D., Kerbel R.S. (2002). Differences in therapeutic indexes of combination metronomic chemotherapy and an anti-VEGFR-2 antibody in multidrug-resistant human breast cancer xenografts. Clin. Cancer Res..

[6] Fouladzadeh A., Dorraki M., Min K.K.M., Cockshell M.P., Thompson E.J., Verjans J.W., Allison A., Bonder C.S., Abbott D. (2021). The development of tumour vascular networks. Commun. Biol..

[7] Natale G., Bocci G. (2018). Does metronomic chemotherapy induce tumor angiogenic dormancy? A review of available preclinical and clinical data. Cancer Lett..

[8] Kerbel R.S., Kamen B.A. (2004). The anti-angiogenic basis of metronomic chemotherapy. Nat. Rev. Cancer.

[9] Vasudev N.S., Reynolds A.R. (2014). Anti-angiogenic therapy for cancer: Current progress, unresolved questions and future directions. Angiogenesis.

[10] Ghosh Dastidar D., Ghosh D., Chakrabarti G. (2020). Tumour vasculature targeted anti-cancer therapy. Vessel Plus.

[11] Di Desidero T., Xu P., Man S., Bocci G., Kerbel R.S. (2015). Potent efficacy of metronomic topotecan and pazopanib combination therapy in preclinical models of primary or late stage metastatic triple-negative breast cancer. Oncotarget.

[12] Andre N., Tsai K., Carre M., Pasquier E. (2017). Metronomic Chemotherapy: Direct Targeting of Cancer Cells after all?. Trends Cancer.

[13] Parra K., Valenzuela P., Lerma N., Gallegos A., Reza L.C., Rodriguez G., Emmenegger U., Di Desidero T., Bocci G., Felder M.S. (2017). Impact of CTLA-4 blockade in conjunction with metronomic chemotherapy on preclinical breast cancer growth. Br. J. Cancer.

[14] Khan K.A., Ponce de Leon J.L., Benguigui M., Xu P., Chow A., Cruz-Munoz W., Man S., Shaked Y., Kerbel R.S. (2020). Immunostimulatory and anti-tumor metronomic cyclophosphamide regimens assessed in primary orthotopic and metastatic murine breast cancer. NPJ Breast Cancer.

[15] Orecchioni S., Talarico G., Labanca V., Calleri A., Mancuso P., Bertolini F. (2018). Vinorelbine, cyclophosphamide and 5-FU effects on the circulating and intratumoural landscape of immune cells improve anti-PD-L1 efficacy in preclinical models of breast cancer and lymphoma. Br. J. Cancer.

[16] Shaked Y., Emmenegger U., Francia G., Chen L., Lee C.R., Man S., Paraghamian A., Ben-David Y., Kerbel R.S. (2005). Low-dose metronomic combined with intermittent bolus-dose cyclophosphamide is an effective long-term chemotherapy treatment strategy. Cancer Res..

[17] Daenen L.G., Shaked Y., Man S., Xu P., Voest E.E., Hoffman R.M., Chaplin D.J., Kerbel R.S. (2009). Low-dose metronomic cyclophosphamide combined with vascular disrupting therapy induces potent antitumor activity in preclinical human tumor xenograft models. Mol. Cancer Ther..

[18] Fukumura D., Kloepper J., Amoozgar Z., Duda D.G., Jain R.K. (2018). Enhancing cancer immunotherapy using antiangiogenics: Opportunities and challenges. Nat. Rev. Clin. Oncol..

[19] Cramarossa G., Lee E.K., Sivanathan L., Georgsdottir S., Lien K., Santos K.D., Chan K., Emmenegger U. (2014). A systematic literature analysis of correlative studies in low-dose metronomic chemotherapy trials. Biomark Med..

[20] Rajasekaran T., Ng Q.S., Tan D.S., Lim W.T., Ang M.K., Toh C.K., Chowbay B., Kanesvaran R., Tan E.H. (2017). Metronomic chemotherapy: A relook at its basis and rationale. Cancer Lett..

[21] Kim H., Lee Y., Lee S., Kim J.G. (2019). Changes in Breast-tumor Blood Flow in Response to Hypercapnia during Chemotherapy with Laser Speckle Flowmetry. Curr. Opt. Photonics.

[22] Filippone M.G., Gaglio D., Bonfanti R., Tucci F.A., Ceccacci E., Pennisi R., Bonanomi M., Jodice G., Tillhon M., Montani F. (2022). CDK12 promotes tumorigenesis but induces vulnerability to therapies inhibiting folate one-carbon metabolism in breast cancer. Nat. Commun..

[23] Roy A., Sarker S., Upadhyay P., Pal A., Adhikary A., Jana K., Ray M. (2018). Methylglyoxal at metronomic doses sensitizes breast cancer cells to doxorubicin and cisplatin causing synergistic induction of programmed cell death and inhibition of stemness. Biochem. Pharmacol..

[24] Salem A.R., Martinez Pulido P., Sanchez F., Sanchez Y., Espanol A.J., Sales M.E. (2020). Effect of low dose metronomic therapy on MCF-7 tumor cells growth and angiogenesis. Role of muscarinic acetylcholine receptors. Int. Immunopharmacol..

[25] Cerrito M.G., De Giorgi M., Pelizzoni D., Bonomo S.M., Digiacomo N., Scagliotti A., Bugarin C., Gaipa G., Grassilli E., Lavitrano M. (2018). Metronomic combination of Vinorelbine and 5Fluorouracil is able to inhibit triple-negative breast cancer cells. Results from the proof-of-concept VICTOR-0 study. Oncotarget.

[26] Sukumar J., Gast K., Quiroga D., Lustberg M., Williams N. (2021). Triple-negative breast cancer: Promising prognostic biomarkers currently in development. Expert Rev. Anticancer Ther..

[27] Chen Q., Xia R., Zheng W., Zhang L., Li P., Sun X., Shi J. (2020). Metronomic paclitaxel improves the efficacy of PD-1 monoclonal antibodies in breast cancer by transforming the tumor immune microenvironment. Am. J. Transl. Res..

[28] Kamoun W.S., Dugast A.S., Suchy J.J., Grabow S., Fulton R.B., Sampson J.F., Luus L., Santiago M., Koshkaryev A., Sun G. (2020). Synergy between EphA2-ILs-DTXp, a Novel EphA2-Targeted Nanoliposomal Taxane, and PD-1 Inhibitors in Preclinical Tumor Models. Mol. Cancer Ther..

[29] Francia G., Man S., Lee C.J., Lee C.R., Xu P., Mossoba M.E., Emmenegger U., Medin J.A., Kerbel R.S. (2009). Comparative impact of trastuzumab and cyclophosphamide on HER-2-positive human breast cancer xenografts. Clin. Cancer Res..

[30] Vergato C., Doshi K.A., Roblyer D., Waxman D.J. (2022). Type-I Interferon Signaling Is Essential for Robust Metronomic Chemo-Immunogenic Tumor Regression in Murine Breast Cancer. Cancer Res. Commun..

[31] Fowler A.M., Mankoff D.A., Joe B.N. (2017). Imaging Neoadjuvant Therapy Response in Breast Cancer. Radiology.

[32] Tabassum S., Tank A., Wang F., Karrobi K., Vergato C., Bigio I.J., Waxman D.J., Roblyer D. (2021). Optical scattering as an early marker of apoptosis during chemotherapy and antiangiogenic therapy in murine models of prostate and breast cancer. Neoplasia.

[33] Shaked Y., Pham E., Hariharan S., Magidey K., Beyar-Katz O., Xu P., Man S., Wu F.T., Miller V., Andrews D. (2016). Evidence Implicating Immunological Host Effects in the Efficacy of Metronomic Low-Dose Chemotherapy. Cancer Res..

[34] Bocci G., Kerbel R.S. (2016). Pharmacokinetics of metronomic chemotherapy: A neglected but crucial aspect. Nat. Rev. Clin. Oncol..

[35] Colleoni M., Gray K.P., Gelber S., Lang I., Thurlimann B., Gianni L., Abdi E.A., Gomez H.L., Linderholm B.K., Puglisi F. (2016). Low-Dose Oral Cyclophosphamide and Methotrexate Maintenance for Hormone Receptor-Negative Early Breast Cancer: International Breast Cancer Study Group Trial 22-00. J. Clin. Oncol..

[36] Gebbia V., Boussen H., Valerio M.R. (2012). Oral metronomic cyclophosphamide with and without methotrexate as palliative treatment for patients with metastatic breast carcinoma. Anticancer Res..

[37] Jung L., Miske A., Indorf A., Nelson K., Gadi V.K., Banda K. (2022). A Retrospective Analysis of Metronomic Cyclophosphamide, Methotrexate, and Fluorouracil (CMF) Versus Docetaxel and Cyclophosphamide (TC) as Adjuvant Treatment in Early Stage, Hormone Receptor Positive, HER2 Negative Breast Cancer. Clin. Breast Cancer.

[38] Mutlu H., Musri F.Y., Artac M., Kargi A., Ozdogan M., Bozcuk H. (2015). Metronomic oral chemotherapy with old agents in patients with heavily treated metastatic breast cancer. J. Cancer Res. Ther..

[39] Lu Q., Lee K., Xu F., Xia W., Zheng Q., Hong R., Jiang K., Zhai Q., Li Y., Shi Y. (2020). Metronomic chemotherapy of cyclophosphamide plus methotrexate for advanced breast cancer: Real-world data analyses and experience of one center. Cancer Commun..

[40] Briasoulis E., Pappas P., Puozzo C., Tolis C., Fountzilas G., Dafni U., Marselos M., Pavlidis N. (2009). Dose-ranging study of metronomic oral vinorelbine in patients with advanced refractory cancer. Clin. Cancer Res..

[41] Krajnak S., Decker T., Schollenberger L., Rose C., Ruckes C., Fehm T., Thomssen C., Harbeck N., Schmidt M. (2021). Phase II study of metronomic treatment with daily oral vinorelbine as first-line chemotherapy in patients with advanced/metastatic HR+/HER2- breast cancer resistant to endocrine therapy: VinoMetro-AGO-B-046. J. Cancer Res. Clin. Oncol..

[42] Montagna E., Palazzo A., Maisonneuve P., Cancello G., Iorfida M., Sciandivasci A., Esposito A., Cardillo A., Mazza M., Munzone E. (2017). Safety and efficacy study of metronomic vinorelbine, cyclophosphamide plus capecitabine in metastatic breast cancer: A phase II trial. Cancer Lett..

[43] Brems-Eskildsen A.S., Linnet S., Dano H., Luczak A., Vestlev P.M., Jakobsen E.H., Neimann J., Jensen C.B., Dongsgaard T., Langkjer S.T. (2021). Metronomic treatment of vinorelbine with oral capecitabine is tolerable in the randomized Phase 2 study XeNa including patients with HER2 non-amplified metastatic breast cancer. Acta Oncol..

[44] Krajnak S., Battista M., Brenner W., Almstedt K., Elger T., Heimes A.S., Hasenburg A., Schmidt M. (2018). Explorative Analysis of Low-Dose Metronomic Chemotherapy with Cyclophosphamide and Methotrexate in a Cohort of Metastatic Breast Cancer Patients. Breast Care.

[45] Cazzaniga M.E., Torri V., Riva F., Porcu L., Cicchiello F., Capici S., Cortinovis D., Digiacomo N., Bidoli P. (2017). Efficacy and safety of vinorelbine-capecitabine oral metronomic combination in elderly metastatic breast cancer patients: VICTOR-1 study. Tumori.

[46] Cazzaniga M.E., Cortesi L., Ferzi A., Scaltriti L., Cicchiello F., Ciccarese M., Della Torre S., Villa F., Giordano M., Verusio C. (2016). Metronomic chemotherapy with oral vinorelbine (mVNR) and capecitabine (mCAPE) in advanced HER2-negative breast cancer patients: Is it a way to optimize disease control? Final results of the VICTOR-2 study. Breast Cancer Res. Treat..

[47] Cazzaniga M.E., Pinotti G., Montagna E., Amoroso D., Berardi R., Butera A., Cagossi K., Cavanna L., Ciccarese M., Cinieri S. (2019). Metronomic chemotherapy for advanced breast cancer patients in the real world practice: Final results of the VICTOR-6 study. Breast.

[48] Montagna E., Pagan E., Cancello G., Sangalli C., Bagnardi V., Munzone E., Sale E.O., Malengo D., Cazzaniga M.E., Negri M. (2022). The prolonged clinical benefit with metronomic chemotherapy (VEX regimen) in metastatic breast cancer patients. Anticancer Drugs.

[49] Mayer E.L., Isakoff S.J., Klement G., Downing S.R., Chen W.Y., Hannagan K., Gelman R., Winer E.P., Burstein H.J. (2012). Combination antiangiogenic therapy in advanced breast cancer: A phase 1 trial of vandetanib, a VEGFR inhibitor, and metronomic chemotherapy, with correlative platelet proteomics. Breast Cancer Res. Treat..

[50] Colleoni M., Orlando L., Sanna G., Rocca A., Maisonneuve P., Peruzzotti G., Ghisini R., Sandri M.T., Zorzino L., Nole F. (2006). Metronomic low-dose oral cyclophosphamide and methotrexate plus or minus thalidomide in metastatic breast cancer: Antitumor activity and biological effects. Ann. Oncol..

[51] Wildiers H., Tryfonidis K., Dal Lago L., Vuylsteke P., Curigliano G., Waters S., Brouwers B., Altintas S., Touati N., Cardoso F. (2018). Pertuzumab and trastuzumab with or without metronomic chemotherapy for older patients with HER2-positive metastatic breast cancer (EORTC 75111-10114): An open-label, randomised, phase 2 trial from the Elderly Task Force/Breast Cancer Group. Lancet. Oncol..

[52] Dal Lago L., Uwimana A.L., Coens C., Vuylsteke P., Curigliano G., Brouwers B., Jagiello-Gruszfeld A., Altintas S., Tryfonidis K., Poncet C. (2022). Health-related quality of life in older patients with HER2+ metastatic breast cancer: Comparing pertuzumab plus trastuzumab with or without metronomic chemotherapy in a randomised open-label phase II clinical trial. J. Geriatr. Oncol..

[53] Wang Z., Liu J., Ma F., Wang J., Luo Y., Fan Y., Yuan P., Zhang P., Li Q., Li Q. (2021). Safety and efficacy study of oral metronomic vinorelbine combined with trastuzumab (mNH) in HER2-positive metastatic breast cancer: A phase II trial. Breast Cancer Res. Treat..

[54] Dellapasqua S., Bertolini F., Bagnardi V., Campagnoli E., Scarano E., Torrisi R., Shaked Y., Mancuso P., Goldhirsch A., Rocca A. (2008). Metronomic cyclophosphamide and capecitabine combined with bevacizumab in advanced breast cancer. J. Clin. Oncol..

[55] Garcia-Saenz J.A., Martin M., Calles A., Bueno C., Rodriguez L., Bobokova J., Custodio A., Casado A., Diaz-Rubio E. (2008). Bevacizumab in combination with metronomic chemotherapy in patients with anthracycline- and taxane-refractory breast cancer. J. Chemother..

[56] Rochlitz C., Bigler M., von Moos R., Bernhard J., Matter-Walstra K., Wicki A., Zaman K., Anchisi S., Kung M., Na K.J. (2016). SAKK 24/09: Safety and tolerability of bevacizumab plus paclitaxel vs. bevacizumab plus metronomic cyclophosphamide and capecitabine as first-line therapy in patients with HER2-negative advanced stage breast cancer—A multicenter, randomized phase III trial. BMC Cancer.

[57] Palazzo A., Dellapasqua S., Munzone E., Bagnardi V., Mazza M., Cancello G., Ghisini R., Iorfida M., Montagna E., Goldhirsch A. (2018). Phase II Trial of Bevacizumab Plus Weekly Paclitaxel, Carboplatin, and Metronomic Cyclophosphamide With or Without Trastuzumab and Endocrine Therapy as Preoperative Treatment of Inflammatory Breast Cancer. Clin. Breast Cancer.

[58] Bertolini F., Paul S., Mancuso P., Monestiroli S., Gobbi A., Shaked Y., Kerbel R.S. (2003). Maximum tolerable dose and low-dose metronomic chemotherapy have opposite effects on the mobilization and viability of circulating endothelial progenitor cells. Cancer Res..

[59] Perroud H.A., Rico M.J., Alasino C.M., Queralt F., Mainetti L.E., Pezzotto S.M., Rozados V.R., Scharovsky O.G. (2013). Safety and therapeutic effect of metronomic chemotherapy with cyclophosphamide and celecoxib in advanced breast cancer patients. Future Oncol..

[60] Perroud H.A., Alasino C.M., Rico M.J., Mainetti L.E., Queralt F., Pezzotto S.M., Rozados V.R., Scharovsky O.G. (2016). Metastatic breast cancer patients treated with low-dose metronomic chemotherapy with cyclophosphamide and celecoxib: Clinical outcomes and biomarkers of response. Cancer Chemother. Pharmacol..

[61] Bazzola L., Foroni C., Andreis D., Zanoni V., Cappelletti M.R., Allevi G., Aguggini S., Strina C., Milani M., Venturini S. (2015). Combination of letrozole, metronomic cyclophosphamide and sorafenib is well-tolerated and shows activity in patients with primary breast cancer. Br. J. Cancer..

[62] Kummar S., Wade J.L., Oza A.M., Sullivan D., Chen A.P., Gandara D.R., Ji J., Kinders R.J., Wang L., Allen D. (2016). Randomized phase II trial of cyclophosphamide and the oral poly (ADP-ribose) polymerase inhibitor veliparib in patients with recurrent, advanced triple-negative breast cancer. Investig. New Drugs.

[63] Anampa J., Chen A., Wright J., Patel M., Pellegrino C., Fehn K., Sparano J.A., Andreopoulou E. (2018). Phase I Trial of Veliparib, a Poly ADP Ribose Polymerase Inhibitor, Plus Metronomic Cyclophosphamide in Metastatic HER2-negative Breast Cancer. Clin. Breast Cancer.

[64] Cazzaniga M.E., Munzone E., Bocci G., Afonso N., Gomez P., Langkjer S., Petru E., Pivot X., Sanchez Rovira P., Wysocki P. (2019). Pan-European Expert Meeting on the Use of Metronomic Chemotherapy in Advanced Breast Cancer Patients: The PENELOPE Project. Adv. Ther..

[65] Lien K., Georgsdottir S., Sivanathan L., Chan K., Emmenegger U. (2013). Low-dose metronomic chemotherapy: A systematic literature analysis. Eur. J. Cancer.

[66] Shaked Y., Emmenegger U., Man S., Cervi D., Bertolini F., Ben-David Y., Kerbel R.S. (2005). Optimal biologic dose of metronomic chemotherapy regimens is associated with maximum antiangiogenic activity. Blood.

[67] Mishra-Kalyani P.S., Amiri Kordestani L., Rivera D.R., Singh H., Ibrahim A., DeClaro R.A., Shen Y., Tang S., Sridhara R., Kluetz P.G. (2022). External control arms in oncology: Current use and future directions. Ann. Oncol..

[68] Grassilli E., Cerrito M.G., Lavitrano M. (2022). BTK, the new kid on the (oncology) block?. Front. Oncol..

[69] Grassilli E., Cerrito M.G., Bonomo S., Giovannoni R., Conconi D., Lavitrano M. (2021). p65BTK Is a Novel Biomarker and Therapeutic Target in Solid Tumors. Front. Cell Dev. Biol..

[70] Kim J.M., Park J., Noh E.M., Song H.K., Kang S.Y., Jung S.H., Kim J.S., Park B.H., Lee Y.R., Youn H.J. (2021). Bruton’s agammaglobulinemia tyrosine kinase (Btk) regulates TPAinduced breast cancer cell invasion via PLCgamma2/PKCbeta/NFkappaB/AP1dependent matrix metalloproteinase9 activation. Oncol. Rep..

[71] Grabinski N., Ewald F. (2014). Ibrutinib (ImbruvicaTM) potently inhibits ErbB receptor phosphorylation and cell viability of ErbB2-positive breast cancer cells. Investig. New Drugs.

[72] Wang X., Wong J., Sevinsky C.J., Kokabee L., Khan F., Sun Y., Conklin D.S. (2016). Bruton’s Tyrosine Kinase Inhibitors Prevent Therapeutic Escape in Breast Cancer Cells. Mol. Cancer Ther..

[73] Varikuti S., Singh B., Volpedo G., Ahirwar D.K., Jha B.K., Saljoughian N., Viana A.G., Verma C., Hamza O., Halsey G. (2020). Ibrutinib treatment inhibits breast cancer progression and metastasis by inducing conversion of myeloid-derived suppressor cells to dendritic cells. Br. J. Cancer..

[74] Kerbel R.S. (2015). A Decade of Experience in Developing Preclinical Models of Advanced- or Early-Stage Spontaneous Metastasis to Study Antiangiogenic Drugs, Metronomic Chemotherapy, and the Tumor Microenvironment. Cancer J..

[75] Scharovsky O.G., Rico M.J., Mainetti L.E., Perroud H.A., Rozados V.R. (2020). Achievements and challenges in the use of metronomics for the treatment of breast cancer. Biochem. Pharmacol..

[76] Krajnak S., Battista M.J., Hasenburg A., Schmidt M. (2022). Metronomic Chemotherapy for Metastatic Breast Cancer. Oncol. Res. Treat..

